# An Oriented Recrystallization Nucleation Mechanism of a Cold-Rolled Pure Ti with Electric-Pulse Treatment

**DOI:** 10.3390/ma17235745

**Published:** 2024-11-24

**Authors:** Qi Shi, Lei Wang, Xiu Song, Yang Liu

**Affiliations:** The Key Laboratory for Anisotropy and Texture of Materials (Ministry of Education), School of Materials Science and Engineering, Northeastern University, Shenyang 110819, China; 1710145@stu.neu.edu.cn (Q.S.); songx@smm.neu.edu.cn (X.S.)

**Keywords:** pure Ti, annealing, electric-pulse treatment, oriented nucleation mechanism, pyramidal dislocation

## Abstract

The effect of electric-pulse treatment (EPT) on the nucleation behavior of a cold-rolled pure Ti was investigated. The specimens are subjected to EPT and then annealed at 650 °C within 10 min. Both the electron backscatter diffraction (EBSD) and transmission electron microscope (TEM) techniques were used for detailing the microstructural evolution of the specimens at the initial stage of recrystallization processing during annealing. The results show that oriented nucleation occurs in the EPTed specimen. The recrystallized grains form in a similar orientation with the deformed matrix grains, and the oriented nucleation originates from the deformed grains with <0001> poles tilted about 20° away from the normal direction (ND20 grains) in the EPTed specimen. Pyramidal <c + a> dislocations could be extensively activated in ND20 grains, while the activated dislocations were mainly on prismatic planes in the other oriented grains. Because the formation of sub-grains cannot be without the pyramidal <c + a> dislocation, oriented recrystallized grains easily form in the EPTed specimen. It is suggested that the increasing of pyramidal dislocation climbing activity is considered the key mechanism of the formation of sub-grains as well as oriented nucleation, resulting from high contents of vacancy induced by EPT.

## 1. Introduction

Pure Ti and its alloys, as light-weight structural material, are often used in fields of transportation and aerospace industries, due to their excellent properties, such as high specific strength, corrosion resistance and low density [1,2,3]. However, the poor formability caused by strong deformation texture, which develops during mechanical processing, is a challenge on the extensive applications [4,5]. Improving the mechanical properties through texture modification is a major area of research. Annealing is found to be an effective way to randomize the deformation texture, which would transform to recrystallization texture upon annealing [6,7,8]. Therefore, clarifying the mechanism of recrystallization is the first step to weaken the texture.

The nucleation process provides a basis for understanding the origin of recrystallization texture. Guan et al. proposed that the orientation of recrystallized grains occurring at the initial stage of recrystallization is maintained during the whole recrystallization process [9,10]. Sebald and Gottstein simulated recrystallization texture evolution under several conditions of nucleation orientation and misorientation distribution and found that nucleation orientation plays an important role in determining the recrystallization texture [11]. Therefore, in the past decades, extensive studies were conducted on hexagonal close-packed (hcp) metals, and nucleation orientation mechanisms have been explored. Random nucleation was reported in Mg alloys, resulting from many factors such as alloying elements and nucleation sites [10,12,13,14]. Some researchers found that the recrystallized grains originating at shear bands, deformation twins and second phase particles have random orientations and weaken the texture [15,16,17,18]. Meanwhile, some reports suggested that the orientations of recrystallized grains scattered from twin orientations and were not random [19,20]. Zhao et al. [21] reported that Mg-Zn-Gd displays oriented nucleation tendency due to a preferred misorientation near 70° <11-20>. It is still not exactly understood how these factors affect the nucleation orientation.

Moreover, most studies of nucleation orientation focused on Mg with a c/a ratio of 1.624, close to the ideal ratio and higher than 1.587 of Ti. The difference between the c/a ratio results in a variation in main slip mode. Both the dislocation storage and recovery can influence nucleation of the recrystallized grains. Investigating the nucleation behavior of Ti can help the complement nucleation mechanism of hcp metals.

EPT has attracted increasing attention as a novel manufacturing method with its energy saving and high efficiency. In recent years, the effects of electric currents on the recrystallization of various metals and alloys, such as Al, Mg, Ti alloys et al. have been studied by many researchers. Zhao et al. found that the recrystallization of hot-rolled 5 wt% SiC/7075Al was promoted by high-frequency EPT [22]. Guo et al. found that recrystallization was accelerated by EPT, and the nucleation rate was increased [23]. Zhao et al. found that a fast recrystallization of a titanium alloy produced by equal channel angular pressing was provoked by EPT [24]. Compared with the conventional heat treatment, assistant recrystallization can be accelerated by EPT, thus the recrystallization mechanism can be changed, and microstructure can be optimized. However, the nucleation orientation mechanism in deformed pure Ti by EPT and annealing has not been clear yet.

Therefore, in the present study, a cold-rolled pure Ti is chosen for investigating the nucleation behavior. The effect of EPT on nucleation is explored. The objective is to unravel the mechanism responsible for the formation of nucleation orientation and the influence of EPT.

## 2. Materials and Methods

The experimental material was obtained from a hot-rolled commercially pure α-Ti sheet with a thickness of 6 mm in a fully annealed state. The sheet was cold-rolled at room temperature with a reduction of 10%. Then, a rectangular plate with a dimension of 10 × 5 × 5.4 mm^3^ was cut from the cold-rolled sheet and subjected to EPT for 15 min. The EPT was performed by an HPC-5 type electric-pulse generator. The electric current density was 128 A/mm^2^ with a duration time of 15 μs and a frequency of 30 Hz for 15 min. During EPT, the direction of the electric-pulse current was along the rolling direction (RD), and the increased temperature was measured to be 70 °C. For comparison, the specimens without EPT were also heated at 70 °C for 15 min. And then both EPTed and non-EPTed specimens were annealed at 650 °C up to 10 min.

The microstructure observation was carried out on a rolling direction–normal direction (RD-ND) section. The EBSD was performed using JSM-7500F scanning electron microscopy (SEM) (JEOL Ltd., Tokyo, Japan) with a step size of 0.1 µm or 1 µm. The small specimens of 8 mm × 3 mm × 5 mm were cut for microstructure observation. The specimens were mechanically grinded by using grit papers. Then, they were electropolished at −20 °C and 0.1 A for 90 s to remove the surface damage. The electrolyte contained concentrations of 60 vol.% methanol, 30 vol.% N-butanol and 10 vol.% perchloric acid. Grain sizes, textures, Schmid factors (SFs) and other microstructural features were acquired from EBSD data using Aztec Crystal software (3.1.391 sp1) [25]. In the EBSD analysis, the grains with a grain orientation spread (GOS) value less than 2° were defined as recrystallized grains. TEM samples were sliced perpendicular to the transverse direction (TD) with a thickness of 500 mm and were mechanically polished to 50 mm. Then, disks with a diameter of 3 mm were punched out and jet electropolished. A TEM observation was utilized with a JEOL 2100 type (JEOL Ltd., Tokyo, Japan).

## 3. Results

### 3.1. Nucleation Behavior

**Figure 1:**
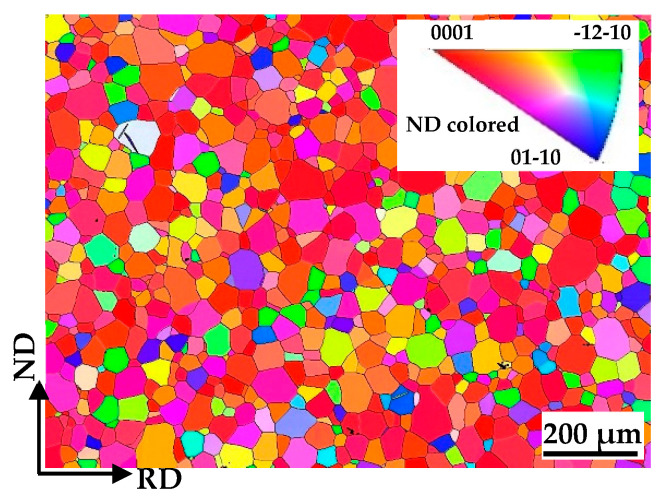
EBSD IPF map of as-received pure Ti.

**Figure 2:**
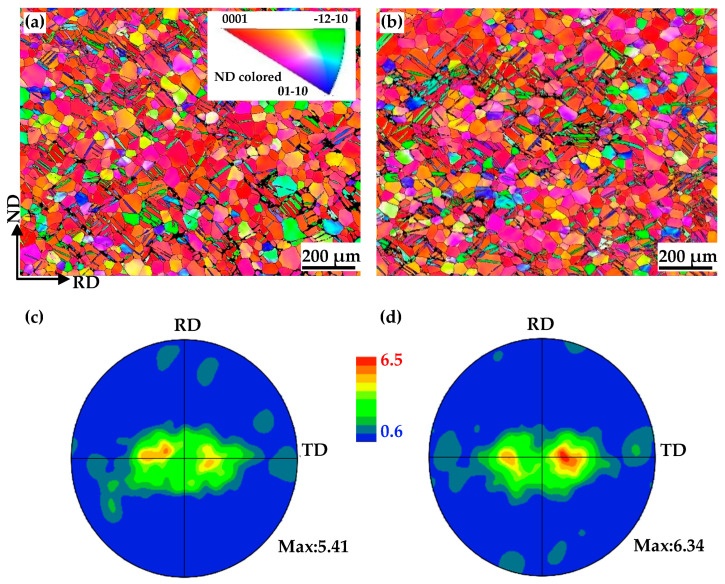
EBSD IPF maps and corresponding (0002) pole figures of (**a**,**c**) non-EPTed specimen; and (**b**,**d**) EPTed specimen.

The as-received microstructure is shown in Figure 1, which shows equiaxed grains with an average grain size of 50 mm. The inverse pole figure (IPF) maps of the non-EPTed and EPTed specimen are shown in Figure 2. It indicates that the grain size and texture were virtually unchanged by EPT. The (0002) pole figure (PF) shown in Figure 2b is a typical TD-split texture, which is normally formed in Ti alloys [24,26], with two separate texture peaks tilted away from the ND to the TD.

**Figure 3:**
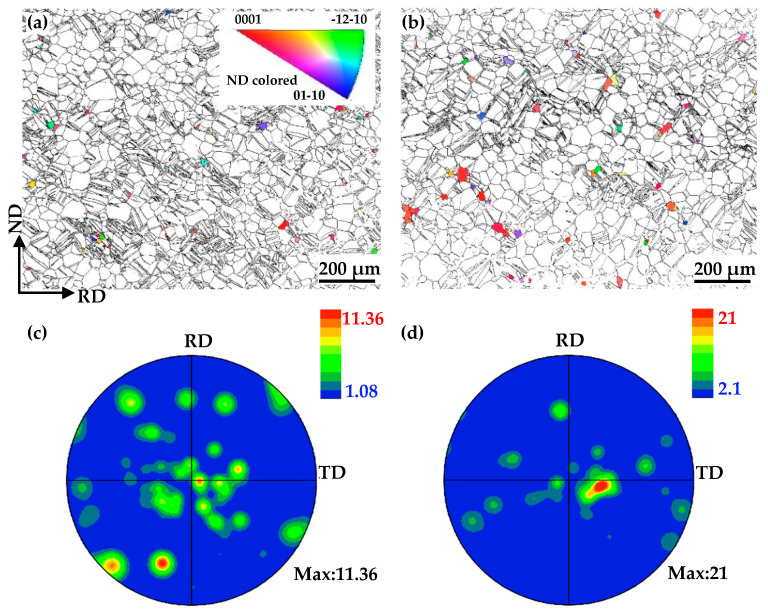
EBSD IPF subset maps and corresponding (0002) pole figures only taken from recrystallized grains of (**a**,**c**) non-EPTed specimen; and (**b**,**d**) EPTed specimen after annealing.

After annealing at 650 °C for 10 min, recrystallized grains can be clearly observed in both non-EPTed and EPTed specimens, as shown in Figure 3. Figure 3a,b shows the IPF subset maps of the recrystallized grains. The recrystallized grains are mostly located at the twin-grain boundary (GB) intersections in the non-EPTed specimen while located at the triple junction of GBs in the EPTed specimen. Figure 3c,d shows the (0002) pole figures of non-EPTed and EPTed specimens only taken from the recrystallized grains, corresponding to Figure 3a,b, respectively. It is evident that various orientated recrystallized grains form in the non-EPTed specimen. However, in the EPTed specimen, the recrystallized grains mostly form tilted about 20° away from the ND, indicating a preferred selection of the recrystallized grain orientations. In other words, the oriented nucleation occurs in the EPTed specimen.

### 3.2. Specific Orientation of Grains

The deformed grains are divided into three groups based on the angle differences between their c-axes with the ND, in order to explore the preferential nucleation at the initial stage of recrystallization in the EPTed specimen. The three groups are 0–20°, 20–50° and 50–90° tilted away from the ND and marked as ND20, ND20–50 and ND50–90, respectively, in the following sections.

As is well known, the stored strain energy of ND20, ND20–50 and ND50–90 grains in both non-EPTed and EPTed specimens can be indicated by the average values of kernel average misorientation (KAM), and the results are listed in Table 1. It is clear that the KAM values of ND20 and ND20–50 grains are similar but lower than those of ND50–90 grains in both non-EPTed and EPTed specimens. This finding suggests that there is a lower stored strain energy in the ND20 and ND20–50 grains. Since the stored strain energy is almost no difference between ND20 grains and ND20–50 grains, the difference between the ND20 and ND50–90 grains is studied.

In order to evaluate the possible activated slip system in grains with different orientations, SFs of basal <a>, prismatic <a>, pyramidal I and II <c + a> are calculated. The average values of the SF of ND20 and ND50–90 grains of EPTed and non-EPTed specimens are shown in Table 2. In ND20 grains, the pyramidal slip system has much higher SFs compared to both prismatic and basal slips, suggesting that ND20 grains are favorable for pyramidal dislocation slip. Meanwhile, in ND50–90 grains, SFs for prismatic and pyramidal dislocations are both high. The type of activated slip system in ND20 and ND50–90 grains can also be further identified by the intragranular misorientation axis (IGMA).

**Figure 4:**
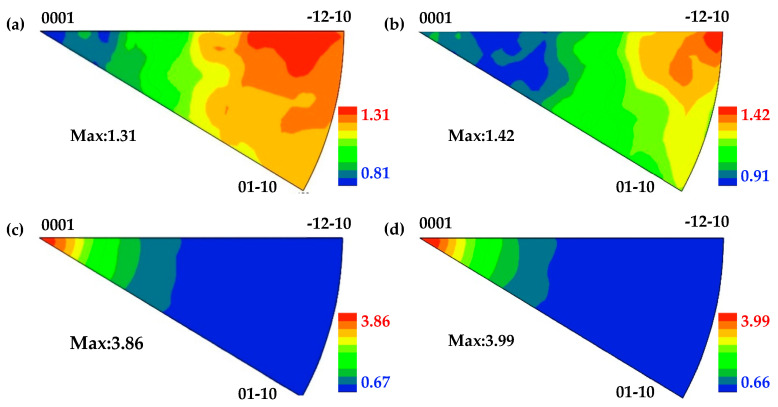
IGMA distribution of (**a**) ND20 grains in non-EPTed specimen; (**b**) ND20 grains in EPTed specimen; (**c**) ND50–90 grains in non-EPTed specimen; and (**d**) ND50–90 grains in EPTed specimen.

The IGMA distribution of ND20 and ND50–90 grains in the non-EPTed and EPTed specimens are shown in Figure 4. The principle of IGMA analysis originates from the lattice rotation around the Taylor axis induced by the action of dislocation [27]. Available slip modes in hcp metals and their respective lattice rotation axes are listed in Table 3 [27]. From Figure 4, intense IGMA peaks are around <0001> with a high peak intensity of 3.86 and 3.99 mud in ND50–90 grains, showing the presence and predominance of the prismatic dislocation slip. In addition, the Taylor axis of pyramidal dislocation is also detected in scatter IGMA data collected from ND50–90 grains, although it does not show any peaks in contour IGMA data. For ND20 grains, IGMA peaks are around <11-20> with a great spread. The peak around <11-20> is usually attributed to the coactivation of basal and pyramidal dislocation slips [28,29]. And the variety of slip mode resulted in the spread. The results are in alignment with the SF prediction.

### 3.3. Dislocation Characteristics

**Figure 5:**
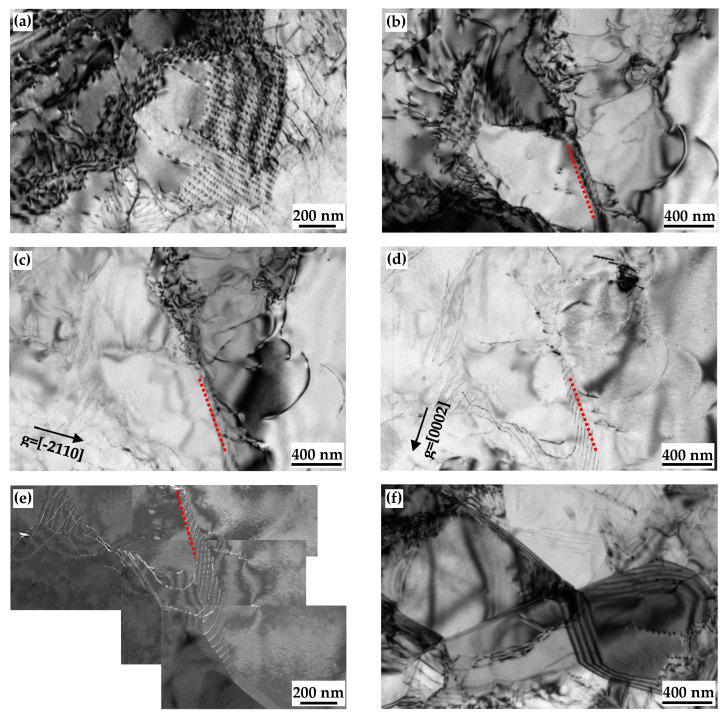
TEM micrographs of EPTed specimen after annealing, showing (**a**) regular array of dislocations; (**b**–**d**) LAGBs; (e) dark field image of LAGB; and (**f**) sub-grains.

Figure 5 shows dislocation configurations at early stages of nucleation in the EPTed specimen. Since the cold-rolling deformation is actually heterogeneous, various substructures simultaneously appear. Figure 5a shows a regular array of dislocations indicating the occurrence of recovery. Some dislocations even rearrange themselves into sub-grains to form low-angle grain boundaries (LAGBs), to reduce the stress concentration, as shown in Figure 5b, and a sub-grain boundary marked with a dotted line forms with the pile up of dislocations. Figure 5e shows sub-grains with thinner and sharper boundaries, which would transform to recrystallized grains.

Figure 5b–e shows two-beam and weak-beam observations of the LAGB under **g** = [-2110] and **g** = [0001] reflection, respectively. Based on the **g•b** criterion, under **g** = [0001] reflection, <a> dislocation should be invisible, and under the **g** = [-2110] reflection, <a> dislocation should be invisible. Only <c + a> dislocations can be seen in both reflections. Clearly the LAGB was visible when **g** = [-2110] and [0001]. Therefore, the LAGB comprises opyramidal <c + a> dislocations.

## 4. Discussion

As described above, there is a wide variety of recrystallized grain orientations generated in the non-EPTed specimen after annealing at 650 °C for 10 min. Meanwhile, oriented nucleation appeared in the EPTed specimens at the same annealing condition. And the recrystallized grains are mostly at the ND20 orientation with <0001> poles tilted about 20° away from the normal direction. The mechanism is discussed as follows.

### 4.1. The Characteristics of Recrystallization Nucleation

Shear bands, GBs, twins and grain junctions are reported to be the main nucleation sites for recrystallization in Ti alloys [30,31,32]. The effect of shear bands on nucleation could be excluded because shear bands cannot nearly form with a cold-rolling reduction of 10%. Recent studies indicated that GBs and twins provide more nucleation sites for recrystallized grains with random orientations [14]. However, in the EPTed specimen, the recrystallized grains originating at the junctions of GBs had a specific orientation of ND20, as shown in Figure 3. It is suggested that the nucleation site made limited contributions to the oriented nucleation in the EPTed specimen.

**Figure 6:**
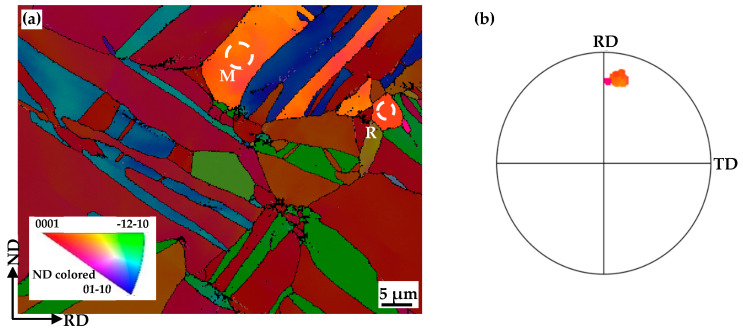
(**a**) EBSD IPF subset map of recrystallized grain (R) and deformed matrix grain (M); (**b**) corresponding (0002) pole figure of EPTed specimen after annealing.

**Figure 7:**
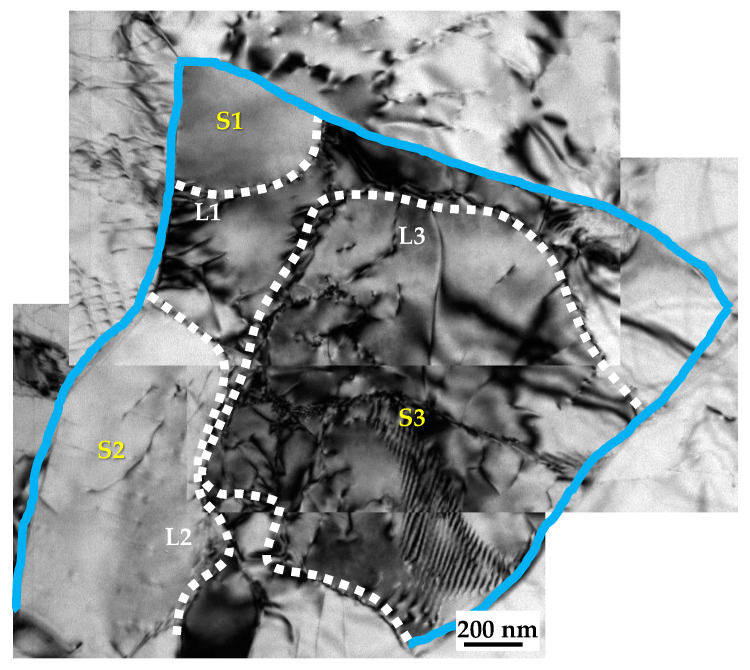
TEM micrographs of EPTed specimen after annealing, showing sub-grains.

**Figure 8:**
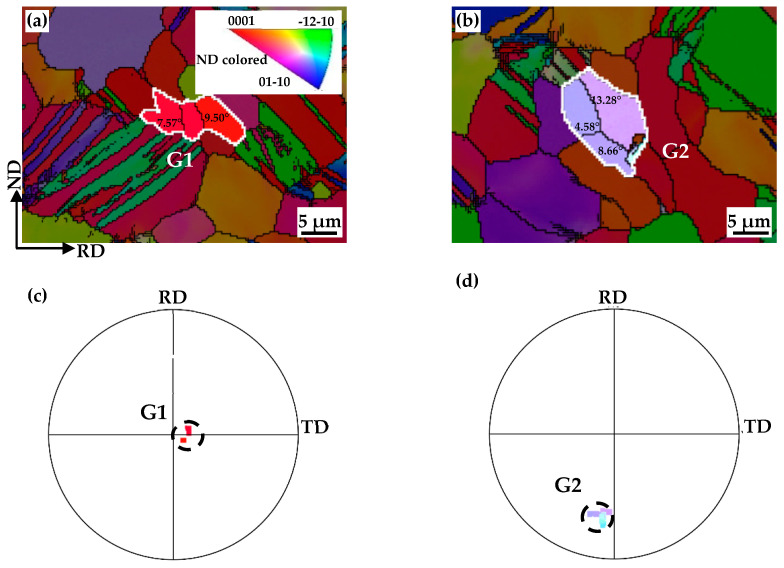
EBSD IPF maps and corresponding (0002) pole figures of recrystallized grains: (**a**,**c**) G1; and (**b**,**d**) G2.

Figure 6 shows that the c-axis of the recrystallized grain (R) is close to its deformed matrix grain (M). Recrystallization nucleation differs from classical phase transformation [33]. The recrystallized grains originate from volumes, which are believed to already exist in the deformed grains [34]. And this nuclei-to-matrix orientation relationship has also been reported in Al, TA15 and cold-rolled Mg-Zn-Gd [35,36,37]. In order to reduce the stress concentration, the tangled dislocations spontaneously rearrange themselves into sub-grains and form low-angle grain boundaries. Figure 7 shows sub-grains (S1, S2 and S3) surrounded by LAGBs (L1, L2 and L3) within a deformed matrix grain (marked with blue lines) of the EPTed specimen after annealing. These sub-grain boundaries would continually absorb dislocations until the dislocations inside the sub-grains are eliminated. And then, clean sub-grains formed with much thinner and sharper boundaries, as shown in Figure 5e. These sub-grains are viable nuclei for the recrystallized grains. Accompanied by this process, misorientation of the sub-grain boundaries increase, and then, these sub-grain boundaries could transform into HAGBs. Thus, the deformed matrix grain would transform to a recrystallized grain containing LAGBs inside, as shown in Figure 8a,b, showing two individual recrystallized grains (G1 and G2) containing LAGBs inside. The different sub-grains separated by these LAGBs have similar orientations, as shown in (0001) the pole figures in Figure 8c,d. It indicates that the recrystallized grains evolving from sub-grains inherit orientations from the deformed matrix grains. Therefore, the oriented nucleation demonstrates that recrystallization preferentially occurred in ND20 grains.

The difference in the nucleation behavior of grains with specific orientations is attributable either to thermodynamics or kinetics. Since ND20 grains had a lower stored strain energy than ND50–90 grains, it is suggested that driving force is not the essential factor. Therefore, the difference can be considered depended on kinetics through the disparity of dislocation activity in ND20 and ND50–90 grains rather than thermodynamics.

The results shown in Figure 4 indicate that prismatic slip dominates in ND50–90 grains, while pyramidal and basal slips are prevalent in ND20 grains. The activation of the slip system is governed by the combined effect of SFs and critical resolved shear stress (CRSS). Although the SF values of the prismatic and pyramidal slip in ND50–90 grains are both high, the CRSS of pyramidal slip is about 3–5 times higher than that of the prismatic slip at room temperature [38,39,40]. It has been found that the prismatic slip is the dominant deformation mode for the grains with equal SFs of basal and prismatic dislocation slips [41]. Thus, the strain in ND50–90 grains predominantly contributed to prismatic dislocations, with few pyramidal dislocations contributing to accommodating the strain along the <c> direction. The SF values of the prismatic slip in ND20 is extremely low, so the activation stress (CRSS/SF) required for activating it is too high. The difference in dislocation activities between ND20 and ND50–90 grains is the key factor as to why recrystallization preferentially occurred in ND20 grains with a lower stored strain energy.

**Figure 9:**
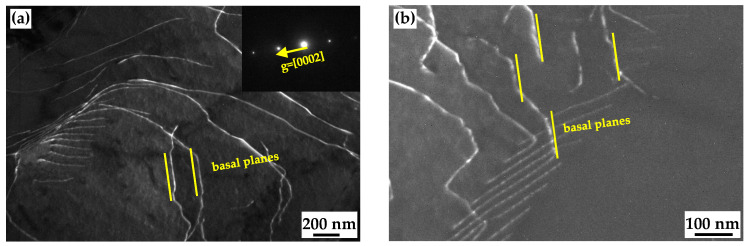
TEM micrographs of EPTed specimen after annealing, showing the dislocations with the <c> component on the basal planes under **g** = [0001].

The pyramidal dislocation plays an essential role in the formation of sub-grains. It is found with TEM observation that the LAGB was composed of pyramidal <c + a> dislocations (as shown in Figure 5). It is also reported that the formation of the LAGB via edge-type dislocations in Mg alloys is crucial to accomplish polygonization [42]. In the present study, the pyramidal dislocations forming the LAGBs are not edge but mixed instead. The mobility of pyramidal dislocation is low. In addition, sessile dislocations with the <c> component on the basal planes were observed, as shown in Figure 9. These dislocations may result from the dislocation decomposition from <c + a> dislocations [43]. The low mobility of pyramidal dislocation hindered further migration of dislocations and facilitated the formation of LAGBs.

**Figure 10:**
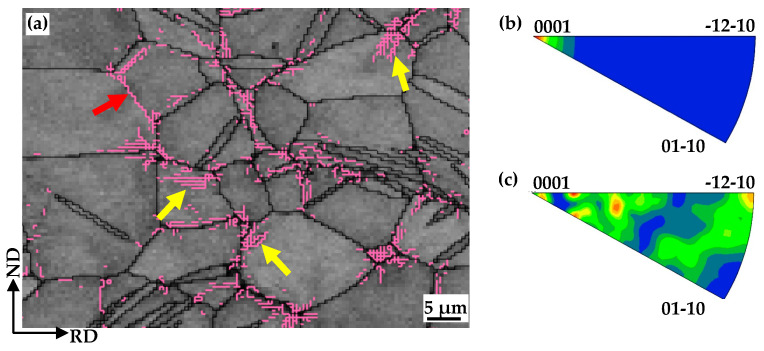
(**a**) Local magnificent band contrast (BC) map superimposed with grain boundary component of EPTed specimen after annealing, showing classification of LAGB; (**b**,**c**) IGMA distribution of different groups of LAGBs.

For statistical reliability, an IGMA analysis was conducted to examine the dislocation type arranged into the LAGBs. Figure 10 shows the classification and IGMA distribution of different groups of LAGBs. Two kinds of LAGBs are indexed in EBSD maps and marked with pink color (Figure 10a): (i) the continued pixel shown by the red arrows; and (ii) the discontinued pixel shown by the yellow arrows. The peak around <0001> shown in Figure 10b demonstrates that the selected discontinued LAGBs form with prismatic dislocations. Meanwhile, a complex distribution of the IGMA of the continued LAGBs is depicted in Figure 10c, which is induced with multiple activations of both basal and pyramidal dislocations. It is suggested that the participation of pyramidal dislocation is indispensable to the formation of continuous LAGBs.

### 4.2. Oriented Nucleation New Mechanism in EPTed Specimen

According to the analysis in Section 4.1., the nucleation of ND20 grains is mainly attributed to the high activity of pyramidal <c + a> dislocations, which is essential to the formation of LAGBs. And it only occurred in the EPTed specimen. Usually, the EPT effects include the thermal effects (the Joule heat) and athermal effects. The Joule heat of the EPTed specimen in the present study produced an increased temperature of 70 °C, which is extremely low relative to the recrystallization temperature of a pure Ti. And the non-EPTed specimen was also heated at 70 °C before annealing at 650 °C to eliminate the influence of Joule heat. Therefore, only the athermal effect of EPT can be taken into account.

It was found that atomic vibration energy could be enhanced, and more vacancies were generated by electrostatic field [44]. Han et al. [45] also proposed that the electropulsing assisted aging processing can enhance the vacancy diffusion coefficient via interaction between drift electrons and vacancies. The flux of atoms in metals induced by EPT is given by [46,47,48,49]:

(1) $$J_{i}=\frac{N_{i}\cdot D_{i}}{kT}(kT\cdot \frac{\partial \ln X_{i}}{\partial x}-\Omega \cdot \frac{\partial \sigma }{\partial x}+Z^{*}\cdot e\cdot \rho \cdot J)$$

where $N_{i}$ is the density of the i atom species, $D_{i}$ is the pertinent diffusion coefficient, k is the Boltzmann constant, T is the absolute temperature, $X_{i}$ is the concentration of the ith solute, Ω is the atom value, $\frac{\partial \sigma }{\partial x}$ is the stress gradient, $Z^{*}$ is the effective valence, e is the charge on an electron, ρ is the resistivity and J is the current density.

As is well known, the absorption or emission of vacancies is the key point for dislocation climbing because of its high activation energy. Dislocation climbing easily occurs with a higher concentration of vacancy. Considering the interaction between vacancy and dislocation in different slip systems, the vacancy concentration nearby the dislocation can be represented as follows [50]:

(2) $$c=c_{0}exp(\frac{-E_{I}}{kT})$$

where $c_{0}$ is the average vacancy concentration, $E_{I}$ is the interaction energy between dislocation and vacancy and can be expressed as follows [51]:

(3) $$E_{I}=\frac{4\left(1+\nu \right)\mu br^{3}\delta }{3(1-\nu )}\frac{x_{2}}{\left(x_{1}^{2}+x_{2}^{2}\right)}$$

where ν is Poisson’s ratio, μ is the shear modulus, δ is the misfit parameter (about −0.1~0 for vacancy), b is the Burgers vector, r is the atomic radius and $x_{1}$ and $x_{2}$ are the coordinate parameters. It can be suggested that the vacancy concentration around {10-11} <11-23> pyramidal dislocations is higher than that around {10-10} <11-20> prismatic dislocations. The mobility of pyramidal dislocation in the EPTed specimen could be improved and oriented nucleation forms.

**Figure 11:**
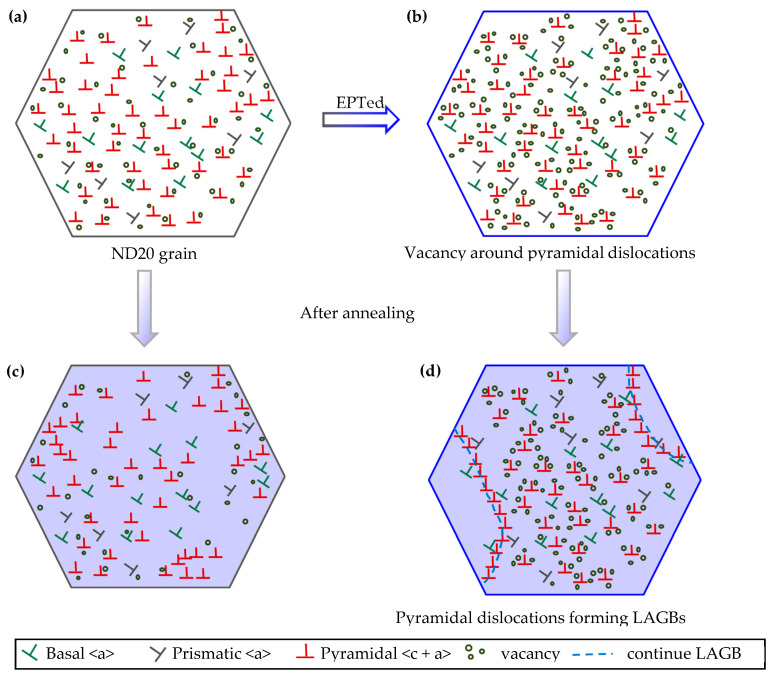
Schematic illustration showing nucleation behavior of ND20 grain in EPTed and non-EPTed specimens. (**a**) ND20 grain in non-EPTed specimen; (**b**) ND20 grain in EPTed specimen; (**c**) Nucleation of ND20 grain in non-EPTed specimen; (**d**) Nucleation of ND20 grain in EPTed specimen.

The oriented nucleation mechanism of the ND20 grain by EPT is shown in Figure 11. Vacancy concentration is increased by EPT, and it is mainly biased around the pyramidal dislocations, as shown in Figure 11b. The mobility of pyramidal dislocation is effectively improved by the excess vacancies, which assist the formation of LAGBs during annealing. Plenty of pyramidal dislocations are activated in the ND20 grain, while the prismatic dislocation slip is the dominant mechanism in the ND50–90 grain. Therefore, nucleation occurs preferentially in the ND20 grain, resulting from the high contents of vacancy induced by EPT.

## 5. Conclusions

The nucleation behavior of cold-rolled pure Ti subjected to EPT and then annealing at 650 °C within 10 min was investigated. The recrystallized grain orientations were detected.

(1) Oriented nucleation occurred in the EPTed cold-rolled pure Ti specimen. The recrystallized grains exhibited ND20 orientation in the EPTed specimen, while the random orientation of nucleation formed in the non-EPTed specimen.
(2) With EPTed, pyramidal <c + a> dislocations could be extensively activated in ND20 grains, while the activated dislocations are mainly on prismatic planes in the other grains. The formation of sub-grains cannot be without the pyramidal <c + a> dislocation; therefore, ND20 grains recrystallized preferentially.
(3) The athermal effect of EPT enhanced the formation of sub-grain boundaries by increasing the vacancy concentration available to assist pyramidal dislocation climbing, further resulting in oriented nucleation.

## Figures and Tables

**Table 1:** Average KAM values of non-EPTed and EPTed specimens (°).

	Non-EPTed	EPTed
ND20	0.63	0.65
ND20–50	0.65	0.63
ND50–90	0.75	0.8

**Table 2:** Average SF values of EPTed/non-EPTed specimens.

Slip Type	Basal	Prismatic	Pyramidal I	Pyramidal II
ND20	0.21/0.21	0.03/0.03	0.48/0.48	0.49/0.49
ND50–90	0.26/0.28	0.42/0.41	0.45/0.44	0.42/0.42

**Table 3:** List of Taylor axes corresponding to deformation modes in α-Ti.

Slip Mode	Slip Type	Number of Slip	Taylor Axis
{0001} <11-20>	Basal <a>	3	<1-100>
{10–10} <1-210>	Prismatic <a>	3	<0001>
{10–11} <11-2-3>	Pyramidal I <c + a>	12	<-25.41, -16.9>
{11–22} <11-2-3>	Pyramidal II <c + a>	6	<-1100>

## Data Availability

The authors can provide details of this research upon request by letter and commenting on their needs.
